# Personal

**Published:** 1911-04

**Authors:** 


### PERSONAL

---

DR. LEE H. SMITH, of Buffalo, examiner in pathology of the New York State Board of Medical Examiners, attended the Twenty-first annual meeting of the National Confederation of State Medical Examining and Licensing Boards held at Chicago, February 28, 1911, as a delegate from the board. He responded to the addresses of welcome on behalf of the Illinios State Board of Health and of the Illinois State Medical Society in place of Dr. William Warren Potter, who was unable to be present. He also opened the discussion on the symposium on state control of medical colleges, the opening paper of which was by Charles William Dabney, President of the University of Cincinnati, his subject being "Could the state control and conduct medical colleges more efficiently than corporations and private individuals?"

---

DR. JOHN J. DRAKE, of Lackawanna, has been appointed a member of the Buffalo board of pension examining surgeons.

---

DR. CHARLES O'REILLY, of Toronto, spent a day in Buffalo recently calling on a number of his many professional friends.

---

DR. BALDWIN MANN, son of E. B. Mann of Buffalo, has been appointed assistant pathologist and bacteriologist at the Roosevelt Hospital, New York.

---

DR. GEORGE B. STOCKER, of Buffalo, has been appointed deputy medical examiner in place of Dr. John D. Howland, resigned

---

DR. R. A. PAXTON, of Buffalo, has recently been appointed medical school examiner by health commissioner Francis E. Fronczak.

---

DR. H. E. HAYD, of Buffalo, returned early in March from a month's trip in the south. He visited Richmond, Savannah, Palm Beach and other places of interest.

Dr. Benjamin H. Grove, of Buffalo, has resigned from the medical staff of the Emergency Hospital, a position he had held from the opening of the hospital some ten years ago.

